# Enhancing computer image recognition with improved image algorithms

**DOI:** 10.1038/s41598-024-64193-3

**Published:** 2024-06-14

**Authors:** Lanqing Huang, Cheng Yao, Lingyan Zhang, Shijian Luo, Fangtian Ying, Weiqiang Ying

**Affiliations:** 1 Zhejiang University, Hangzhou, 310027 China; 2 Macau University of Science and Technology, Macau, 519020 China

**Keywords:** Improving image, Optimizing the methods, Image recognition, Visual data, Feature extraction, Engineering, Mathematics and computing

## Abstract

Advances in computer image recognition have significantly impacted many industries, including healthcare, security and autonomous systems. This paper aims to explore the potential of improving image algorithms to enhance computer image recognition. Specifically, we will focus on regression methods as a means to improve the accuracy and efficiency of identifying images. In this study, we will analyze various regression techniques and their applications in computer image recognition, as well as the resulting performance improvements through detailed examples and data analysis. This paper deals with the problems related to visual image processing in outdoor unstructured environment. Finally, the heterogeneous patterns are converted into the same pattern, and the heterogeneous patterns are extracted from the fusion features of data modes. The simulation results show that the perception ability and recognition ability of outdoor image recognition in complex environment are improved.

## Introduction

Computer image recognition, a sub-set of computer vision, aims to emulate the remarkable capability of the human visual system. The technique enables machines to recognize and classify objects, faces, scenes, and activities based on intricate image features and patterns^1^. Robot vision is one of the fields where this technology finds its application. As the related technologies are advancing, there is an increasing demand for the classification of a wide range of objects as well as the identification of complex structures^2^. Various applications of image recognition technology are evident in numerous domains, such as traffic management systems, medicine, botany, and meteorology, to name a few. In traffic management systems, automatic license plate recognition is implemented to register illegal vehicles. Within the field of medicine, analyzing cellular or organ shape and color in medical images assists in determining the presence of lesions. In botany, recognition of features like shape and color is helpful for determining the optimal timings of plant watering and fertilization. Additionally, in meteorology, weather forecasts are made possible by observing and calculating satellite imagery^3^. Image recognition technology has become increasingly prevalent in our daily lives. For instance, the facial recognition system is utilized at station entrances to compare passengers’ ID photos, aiding in their identification. Mobile phone fingerprint locks have become more common as well^4^. Accurate identification and detection of images is of utmost importance. Over the past 50 years, computer image recognition technology has experienced a rapid development. Despite massive strides in the field of computer vision, the accurate and fast recognition of images, especially in uncontrolled environments, remains a major challenge. Many traditional image recognition algorithms are limited in their ability to handle variations in scale, orientation, illumination, and viewpoint. They also often struggle with classification tasks when cluttered or occluded objects are present in images^5^. To overcome these challenges, researchers have turned to more advanced image processing techniques. Among them, regression methods have shown significant promise in enhancing the performance of image recognition systems. Regression methods, which originated from the field of statistics, involve predicting a continuous output variable based on input features. In the context of image recognition, regression techniques can be used to learn the mapping from image features to the categories they represent^6^. A novel texture descriptor, MT-ULTP, for cell phenotype classification in fluorescence microscope images, outperforming existing texture descriptors and emphasizing the importance of considering uniform textural patterns in image analysis^7^.Nasim Kayhan^8^ proposes a new approach for content-based image retrieval using a weighted combination of color and texture features, outperforming state-of-the-art methods in terms of precision and recall rate, based on experiments conducted on the Corel 1K and Corel 10K datasets. Deep learning and computer vision, particularly YOLO 7 and TensorFlow 2.8.0, as presented by Sarswat et al., offer an innovative solution that enhances the economic and environmental value of e-waste recycling by accurately categorizing and sorting its components with high precision and recall rates, as well as real-time detection capabilities^9^. Shukhratov et al.^10^ have proposed an Internet of Video Things solution grounded in deep learning algorithms, integrating state-of-the-art object detection models such as Faster R-CNN, RetinaNet, and YOLOv8 onto embedded systems, complemented by the application of quantization techniques to achieve expedited processing times on commercial off-the-shelf embedded platforms. The system attained a high mean Average Precision (mAP) of 77.74% and an accuracy of 95.67% on a test dataset, with further fine-tuning and optimization for deployment on Nano embedded systems, delivering a processing capability of 20 frames per second. This advancement significantly enhances the precision and efficiency of image recognition and classification for plastic waste, particularly PET and PP, on moving conveyor belts, offering an efficacious technological approach to industrial waste management and recycling practices. Gao et al. have introduced an efficient multi-label image classification model for the identification of minerals in Earth sciences^11^, utilizing a dataset of mineral photographs simulated in real-world environments to create realistic feature datasets. The model employs the Query2Label framework, with MaxViT-T serving as the feature extraction network and an asymmetric loss function. Additionally, it incorporates knowledge distillation to enhance identification accuracy and mitigate computational complexity. This approach has achieved state-of-the-art recognition accuracy, surpassing both traditional methods and existing mineral identification models, while maintaining a lower parameter count and computational complexity. Ultimately, ablation studies have substantiated the efficacy of each optimization strategy employed. Singh et al. have demonstrated that employing real-time object recognition algorithms such as YOLO 7 and 5, in conjunction with TensorFlow 2.8.0^12^, these deep learning and computer vision-driven methodologies can efficiently categorize and sort electronic waste components. Particularly, these methods have achieved high F1 scores and mean average precision for materials like copper and printed circuit boards (PCBs), thereby introducing significant financial and environmental benefits to the e-waste recycling process and enhancing its efficiency. Singh et al.^13^have introduced an automated wood diameter level sorting system that integrates computer vision technology for real-time diameter measurement and a deep learning-based binocular vision measurement method, enhancing the efficiency, accuracy, and reducing the labor and cost associated with traditional manual wood sorting processes, thereby significantly advancing the wood production and manufacturing industry towards an automated era.

Taking into consideration this background information, the paper integrates an improved image algorithm in order to establish a computer image recognition system. The image classification and network recognition system utilize ResNet34 as the fundamental structure, and the respective network structure is enhanced and optimized for recognition-related tasks. The fixed input size problem is resolved by replacing the fully connected layers with 1 $\times$ 1 convolutions. If the network’s scale increases, there is a high likelihood of over-fitting, which can be attributed to the majority of weight parameters of the fully connected layer. Consequently, over-fitting measures, such as regularization, ought to be taken to prevent it. If the 1 $\times$ 1 convolution form is used, there are lesser weight parameters generated. The proposed algorithmic improvements in this paper are effective in computer image recognition systems. The effort is to highlight the potential of these methods in not just improving image algorithms, but also in enhancing the efficiency and accuracy of image recognition systems in real-world, practical applications.

## Related work

The literature compares traditional machine learning methods against convolutional neural network-assisted image recognition and classification, concluding that existing methods pose several issues. Traditional machine learning is limited in terms of accuracy and requires thresholding. Moreover, manual extraction of image features is necessary^14^. Interestingly, these techniques have seen wide applications across the globe. Deep learning offers new ideas, but it also has problems, such as the inability to build network structures based on medical image features and poor model generalization. To solve these problems, we propose an optimized convolutional neural network model and introduce adaptive dropout depth computing into the model. This method shows good results in the processing of ultrasonic tomography images and the segmentation of lumbar CT medical images, and has high adaptive segmentation ability. This provides a new perspective for medical image segmentation^15^. The literature chose the VGG16 model as the convolutional neural network for image recognition. The traditional machine learning models were improved by employing gradient-growing tree models. Preprocessing was set up for the image datasets to enhance the preprocessed images. Additionally, an improved version of the VGG16 model (which was originally used in the 2014 ILSVRC challenge) was trained^16^. The introduction of K-Means++ algorithm for data preprocessing, the use of improved bidirectional feature pyramid network structure feature fusion, the use of EIoU loss function to optimize boundary box regression, and the introduction of channel attention mechanism in the convolution unit effectively improve the accuracy of ship detection, and has good robustness and generalization ability. Experiments show that the accuracy of this method is 96.1%, which provides a new solution for the analysis and application of remote sensing images^17^. Through model training on different data sets, the results show that the measurement results generated by the model trained with RootPainter are strongly correlated with the manual measurement results, and the model accuracy is proportional to the annotation time. This shows that the deep learning model can be trained with high precision in a relatively short time, and the RootPainter can complete the annotation, training, and processing of the data in one day^18^. The five groups of models are ultimately classified utilizing either a weighted voting algorithm or a majority voting algorithm. The results are then combined to determine the final output as the overall model for classifying the input images. Furthermore, a classifier screening system was established to validate the aforementioned weighted voting algorithm and majority voting algorithm, resulting in significant time savings during the training process^19^. A data enhancement technique is proposed to process image data sets for tool wear classification tasks. By combining synthetic image and transfer learning models, as well as methods utilizing basic image processing and different types of generative adversarial networks, it is possible to improve the classification accuracy of the model by up to 18% when changing the number of available images in the training dataset from 160 to 4800^20^. Pan, B., et al. proposed a new semantic segmentation network for directly labeling each pixel in a hyperspectral image classification task, instead of treating it as a patch image classification problem. By introducing extended convolution, extended Semantic segmentation Network (DSSNet) is created to solve the problem of low spatial resolution of hyperspectral images. This model is designed for hyperspectral image classification, avoids complex pre - and post-processing operations, and shows excellent performance on two common datasets^21^. Soltis, P.S., et al., discussed the application of machine learning and neural networks (such as deep learning) in fields such as plant science.Ying, W. has contributed to the field of computer image recognition technology by developing a computer vision system based on an enhanced image algorithm. This system is capable of classifying, training, and testing photographic images, thereby improving the learning and training efficacy of data derived from original image processing. The advancement leads to increased convenience in people’s lives, reduced labor expenditure, and enhanced production efficiency, propelling the progression of the computer image recognition domain^22^. Advances are mainly driven by improvements in computing infrastructure, increased computing power, improvements in big data management capabilities, and the development of algorithms. Machine learning has been widely used for tasks such as species identification, plant distribution modeling, weed detection, gene expression analysis, and has been used in comparative genomics and high-throughput phenotypic analysis. The application of these new techniques to plant specimen images is expected to revolutionize the study of plant phenology and functional traits^23^. Kyung, W.-J., et al., introduced a color correction algorithm for color images based on multi-scale gray world algorithm, which is used to enhance faded color images. In this method, the coefficients of each filtered image are calculated by the local processing method of multi-scale filtering, and then the coefficients are integrated to calculate the correction ratio of red and blue channels. Finally, the integral coefficient is applied to the gray-scale world algorithm to get the corrected image. Compared to previous methods, the new algorithm performs well in reproducing the corrected colors of both fully and partially faded images, while also enhancing the visibility of the input image^24^. Hameed introduces an improved unsupervised learning technique, the self-organizing mapping algorithm PLSOM2, which realizes the adaptive adjustment of the neighborhood size by introducing the convergence process of the dynamic neighborhood function to accelerate the algorithm. This improvement can effectively suppress the map distortion, improve the convergence of the algorithm, enhance its adaptability and topological preservation. Extensive experimental results prove the consistency and robustness of the method, and point out that the improvement has important application value in practice^25^. This section discusses the species identification system for down, which has been designed to be interactive and easy to use. The system incorporates both automatic and semi-automatic identification technologies, with automatic identification being the primary focus. The study explores the link between human brain structure and cognitive processes, revealing that brain structure directly impacts visual cognitive processes^26^. To begin with, our study delves into the developmental process of human visual cognition, revealing that the human visual system’s initial ability is to discriminate color areas. Furthermore, we have enhanced the concept of synthesizing the Gestalt theory and topological perception theory to better address the classic problem of “object and background segmentation.” This approach has resulted in the refinement of the “saliency-selection-gestalt” visual problem processing strategy^27^.

## Computer image processing algorithms

### Theoretical basis

Computer image processing involves the use of a computer to receive and extract target information. The extracted data is then further processed and classified to enable identification, and subsequently stored and displayed on the target system. Following this initial stage, additional tasks such as image enhancement and compression, restoration, and separation can be performed to meet user requirements. Computer image recognition strives to improve the clarity of images that are distorted by interference and contrast issues caused by long-distance transmission or other factors. This is accomplished by using specific methods to eliminate these issues and enhance the image, allowing the information within to become more easily understood. Due to the computer’s inherent limitations, it is unable to recognize the original image, so it must first be converted into a digital format that the computer can process. This necessitates digital processing, which is a crucial aspect of image processing. To accomplish this, the image must be quantized, undergo grayscale sampling, and other processes to make it more accessible to the computer. These processes occur within the spatial network. In this work, we use the internal clustering validation method to calculate the efficiency of the algorithm by referring to the mean square error (MSE)^28^. Typically, MSE is used to assess the degree of distortion between the original image and the resulting image. For color images, we extend the formula to include the following three components as shown in Eq. (1):

1 $$\begin{aligned} MSE=\frac{1}{3\cdot M\cdot N}\displaystyle \sum _{i=1}^{M}\displaystyle \sum _{j=1}^{N}((R_{ij}-{R}^{\prime }_{ij})^2+(G_{ij}-{G}^{\prime }_{ij} )^2+(B_{ij}-{B}^{\prime }_{ij})^2) \end{aligned}$$

where M is the number of rows in the image, N is the number of columns in the image, $R_{ij}, G_{ij}, B_{ij}$ are the RGB values of the pixel at position (*i*, *j*) in the original image, ${R}^{\prime }_{ij},{G}^{\prime }, {B}^{\prime }_{ij}$ are the RGB values of the pixel at position (*i*, *j*) in the resulted image.

### Image recognition model

To describe regions accurately, it’s important to identify them first. One commonly used approach is to mark regions or boundaries with integers,a process known as labeling or coloring. This method, also known as connected component labeling, assigns a unique label to each region. To determine the total number of regions, you can count the ID numbers, with the largest ID representing the total number of regions. An alternative method is to use a small number of labels and ensure that the labels of adjacent regions are different. However, this approach requires additional pixel information to index the entire region and a separate data structure to store the information. Define the image as R and divide the image into m independent (disjoint) regions Ri as shown in Eq. (2):

2 $$\begin{aligned} R_b^c=\displaystyle \bigcup _{i=1,i\ne b}^{m}R_i \end{aligned}$$

**Figure 1:**
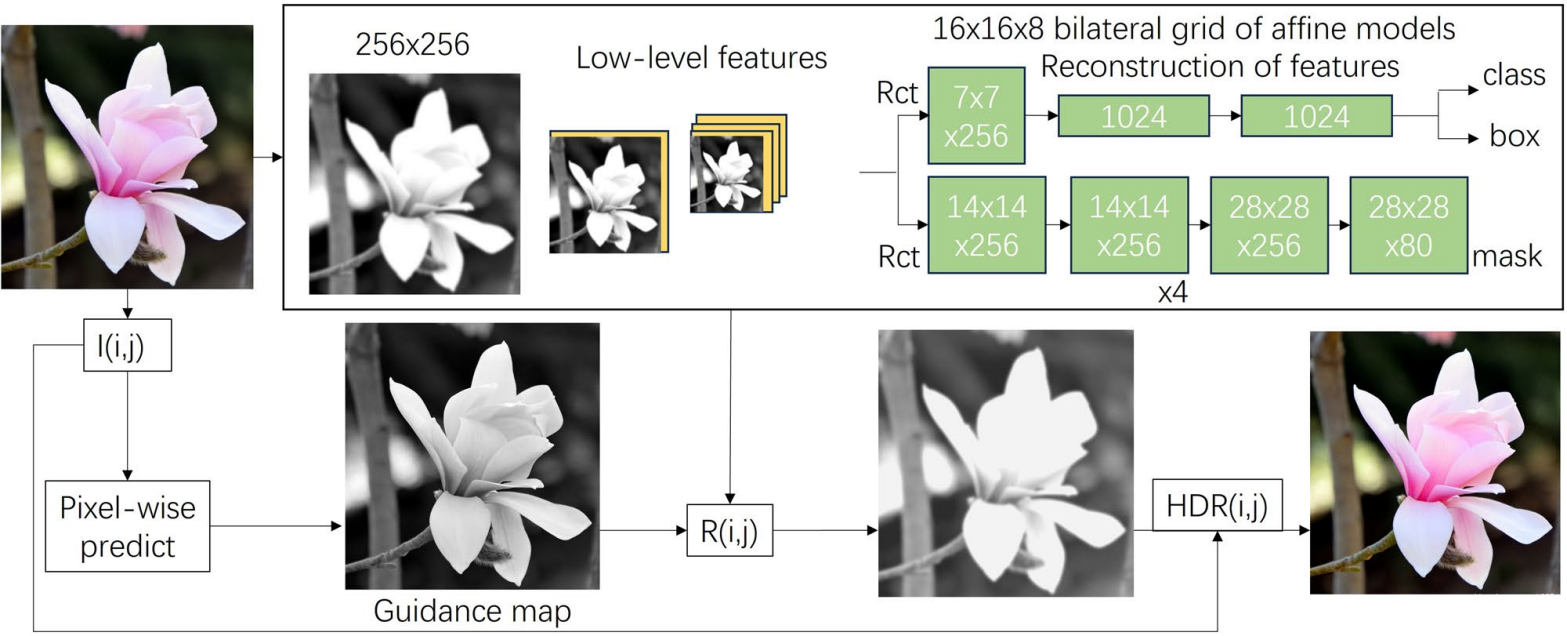
The flow frame diagram of a theoretical model.

The Gaussian filter is a particular kind of linear smoothing filter that is commonly used for removing Gaussian noise.This filter works by scanning each pixel and replacing it with a weighted average of the pixels in the surrounding field of the convolution mask.The center pixel of the mask is then updated with this new weighted value.The discrete convolution formula used to calculate this smoothing effect is Eq. (3):

3 $$\begin{aligned} G(i,j) = \frac{1}{\displaystyle \sum _{k=-m}^{m}\displaystyle \sum _{l=-n}^{n} \exp \left( - \frac{k^2+l^2}{2\sigma ^2}\right) } \displaystyle \sum _{k= -m}^{m}\displaystyle \sum _{l=-n}^{n}\exp \left( -\frac{k^2+l^2}{2\sigma ^2}\right) F(i+k,j+l) \end{aligned}$$

where G (i,j) is the value of the filtered pixel at position (i,j), F (i + k, j + l) is the value of the original pixel at position (i + k, j + l), $\sigma$ is the standard deviation of the Gaussian distribution, m and n are the size of the convolution mask (typically m = n) for square masks.The summation is performed over all pixels in the convolution mask, see Fig. 1.

One approach to obtain high-frequency components is by indirectly extracting them. This involves removing the low-frequency components from the original image, thereby leaving behind the high-frequency components.

4 $$\begin{aligned} E(i,j)=I(i,j)-G(i,j) \end{aligned}$$

Through deep learning technology, LDR images are converted into HDR images, so as to realize dynamic range enhancement and reconstruction of images. By training neural networks to learn image features and reconstruction methods, HDRnet is able to improve the dynamic range of images while preserving details, producing more realistic and natural images. The formula is shown in equation:

1. Input image preprocessing:
  5 $$\begin{aligned} L(i,j)=log(I(i,j)+\varepsilon ) \end{aligned}$$
  where E(i,j) is the correction signal at position (i,j). L(i,j) is the pixel value of the image after preprocessing, I(i,j) is the value of the pixel in the input image at position (i,j). $\varepsilon$ is a small positive number to avoid the zero-value problem in log calculations.
2. Image processing of low dynamic range (LDR) images, feature extraction:
  6 $$\begin{aligned} H(i,j)=Convolution(L(i,j)) \end{aligned}$$
  One of the processed feature images is denoted as H(i,j). The term “Convolution” represents the convolution operation. Feature fusion is the process of combining these features:
  7 $$\begin{aligned} F(i,j)=H(i,j)+L(i,j)+kE(i,j) \end{aligned}$$
  where F(i,j) represents the merged feature image. k is the scaling factor that determines the strength of the enhancement. Feature reconstruction:
  8 $$\begin{aligned} R(i,j)=Deconvolution(F(i,j)) \end{aligned}$$
  where R(i,j) represents the reconstructed LDR image, and Deconvolution signifies the process of performing deconvolution operation.
3. High dynamic range (HDR) image reconstruction, inverse logarithmic transformation:
  9 $$\begin{aligned} HDR(i,j)=exp(R(i,i)) \end{aligned}$$
  Where HDR(i,j) is the reconstructed HDR image.

## Simulation experiment

The training is performed on the original photos, and the results are shown in Table 1.

**Table 1:** Results of training the original data with the improved image algorithm.

Batch size	Accuracy	Precision	Recall	Fl score
16	0.5765	0.4823	0.2515	0.3669
24	0.6814	0.4568	0.4823	0.46955
32	0.6925	0.4175	0.2925	0.355
40	0.74395	0.66265	0.54765	0.60515
48	0.7305	0.55125	0.4355	0.493375
56	0.69805	0.4826	0.35805	0.420325
64	0.7036	0.532	0.36415	0.448075
72	0.7407	0.56	0.441	0.492

**Figure 2:**
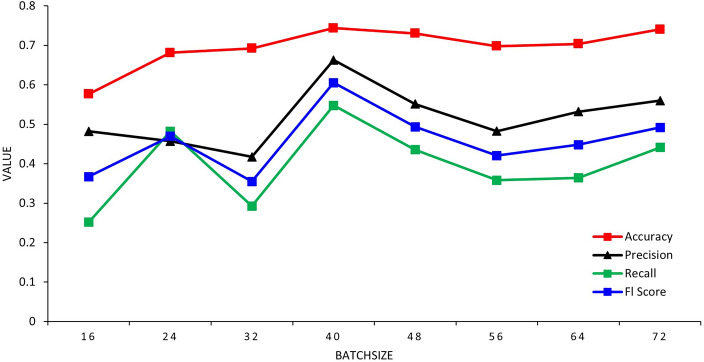
The result of training the original data with the improved image algorithm.

The result of training the original data with the improved image algorithm is shown in Fig. 2:

The training accuracy of the original photo can be observed to be approximately 50%. With an increase in the number of batches, the accuracy increases, but there is a relatively significant fluctuation.

## Improvement and test analysis of computer image recognition algorithm

To improve the algorithm, various methods can be implemented, such as enhancing the preprocessing stage to improve image quality and removing noise. Advanced feature extraction techniques like edge detection or shape recognition can be used to extract relevant information from the images. Training the algorithm using machine learning techniques like deep learning or support vector machines can further enhance its performance. Additionally, thorough testing and analysis should be conducted to evaluate the algorithm’s accuracy and reliability. This involves assessing its performance on different datasets and under various conditions. Overall, improving and analyzing computer image recognition algorithms involves refining preprocessing techniques, applying advanced feature extraction methods, utilizing machine learning, and conducting comprehensive testing.

### Image feature extraction

The weighted voting algorithm combines the classification rates of each branch model. Upon the branch model calculating the classification category, it provides the corresponding classification probability matrix for each category, such as [0.2385, 0.6240, 0.9344, 0.3277, 0.5234], which represents the probability matrix output of one of the modes in the classification. This, in turn, predicts that the input image corresponds to the third label with the highest probability. After training each branch model on the input image, the corresponding recognition accuracy of each model is obtained. The integration idea involves identifying the first word in the final classification of the branch model with high accuracy and the second word in the final classification of the branch model with low accuracy. Assuming that there are three branch modes, the recognition accuracy of mode 1 is $q_1$, the recognition accuracy of mode 2 is $q_2$, and the recognition accuracy of mode 3 is $q_3$. Finally, the weighted voting algorithm is used to combine the classification results of the three models to obtain the weight $w_i$ of the entire model, as shown in Eq. (10):

10 $$\begin{aligned} w_i=\frac{q_1^2+q_2^2+q_3^2}{q_1+q_2+q_3} \end{aligned}$$

11 $$\begin{aligned} Vote(x)=arg\max _{c}{\displaystyle \sum _{i=1}^{n} w_i \cdot f_i (x)} \end{aligned}$$

**Figure 3:**
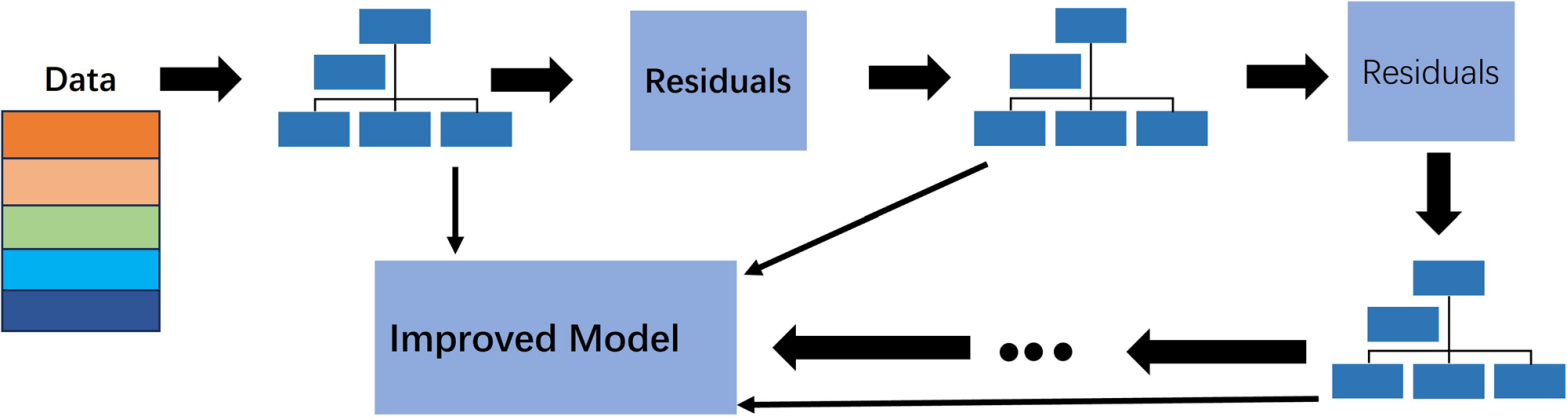
Data structure of majority voting method.

Where Vote(x) is the final result, representing the label with the largest probability of being classified as class c. n is the number of branch models, wi is the weight of the ith branch model, and $f_i(x)$ is the prediction result of the ith model for input x,as shown in Eq. (11). The gradient boosting tree model imported from the external Python library sklearn package is represented by GBDT_CLF. The predic_proba interface function can be called to obtain the classification result calculated by the gradient model. The weighted voting algorithm and the majority voting algorithm differ in that the majority voting algorithm only requires each branch model to provide the final classification result. On the other hand, the weighted voting algorithm surpasses the majority voting algorithm in terms of calculation speed and time consumption as it requires the estimation of the entire probability matrix. The data structure depicted in Fig. 3 is utilized by the majority voting algorithm.

This paper’s model efficacy is demonstrated through a comparison of its precision, recall rate, and calculated F value with those of the CheXNet model^29^. The CheXNet Average model performs with an accuracy, recall, and F-value of 0.387, 0.486, and 0.435 respectively. The average performance in terms of accuracy, recall, and F-value is 0.419, 0.501, and 0.457 respectively.

###  Improve the model structure

The accuracy of image recognition is continually improving. However, increasing the depth of network models can lead to higher memory consumption, greater storage space requirements, and an increase in the number of parameters. There are several common methods for improving network performance: (1) scaling the network’s width or depth while using convolution kernels of different sizes to capture features of varying scales; (2) introducing learning smoothness and generalization through the feature reuse concept of DenseNet; (3) applying the group convolution idea of ResNeXt, where experiments have shown that increasing the number of group convolution groups can enhance performance more effectively than scaling network width and depth; and (4) leveraging channel mixing techniques in ShuffleNet to improve the network’s ability to encode more information without increasing computational complexity. When computing resources are limited, it’s worth considering methods for reducing network parameters and resource consumption without deepening the network. To this end, the convolutional layers and aggregation methods may be improved. In keeping with this approach, an upgraded version of the ResNet34 network is proposed. This involves altering the pooling method and fully connected layer structure, as well as substituting the fully connected layer with 1 $\times$ 1 convolution. The fully connected layer synthesizes global information, extracts partial information from the entire image, and reduces it to a one-dimensional matrix. In contrast, the convolutional layer extracts features within a specific area and applies the convolution kernel to the entire image. Hence, the convolutional layer’s function is similar to that of the fully connected layer, with an equivalent calculation process. By using a 1 $\times$ 1 convolution, the number of convolution kernels in the feature map can be easily controlled. This results in a reduction of parameters due to a corresponding reduction in the number of convolution kernels in the feature map. By replacing fully connected layers with 1 $\times$ 1 convolutions, the fixed input size problem can be addressed. In large networks, most of the weighting parameters tend to accumulate in the fully connected layer, which can lead to overfitting. To prevent this, regularization techniques (e.g., overfitting prevention measures) must be employed. However, the use of 1 $\times$ 1 convolutions circumvents this issue, as it doesn’t generate a significant number of weight parameters. A comparative experiment was conducted to evaluate the impact of network structure on recognition accuracy, using 18 self-built grassland vegetation datasets labeled across 18 distinct classes for training. The experiment will use the same software and hardware environment as previous ones, with 15 iterative training processes employing stochastic gradient descent method and ReLU activation function for random initialization. The initial learning rate will be set to 0.001 and automatically adjusted during training, with a momentum of 0.9, weight parameter decay of 0.0005, and batch size of 8. Ultimately, the experiment aims to determine the extent of network structure’s influence on recognition accuracy through performance comparison before and after implementing changes.

**Figure 4:**
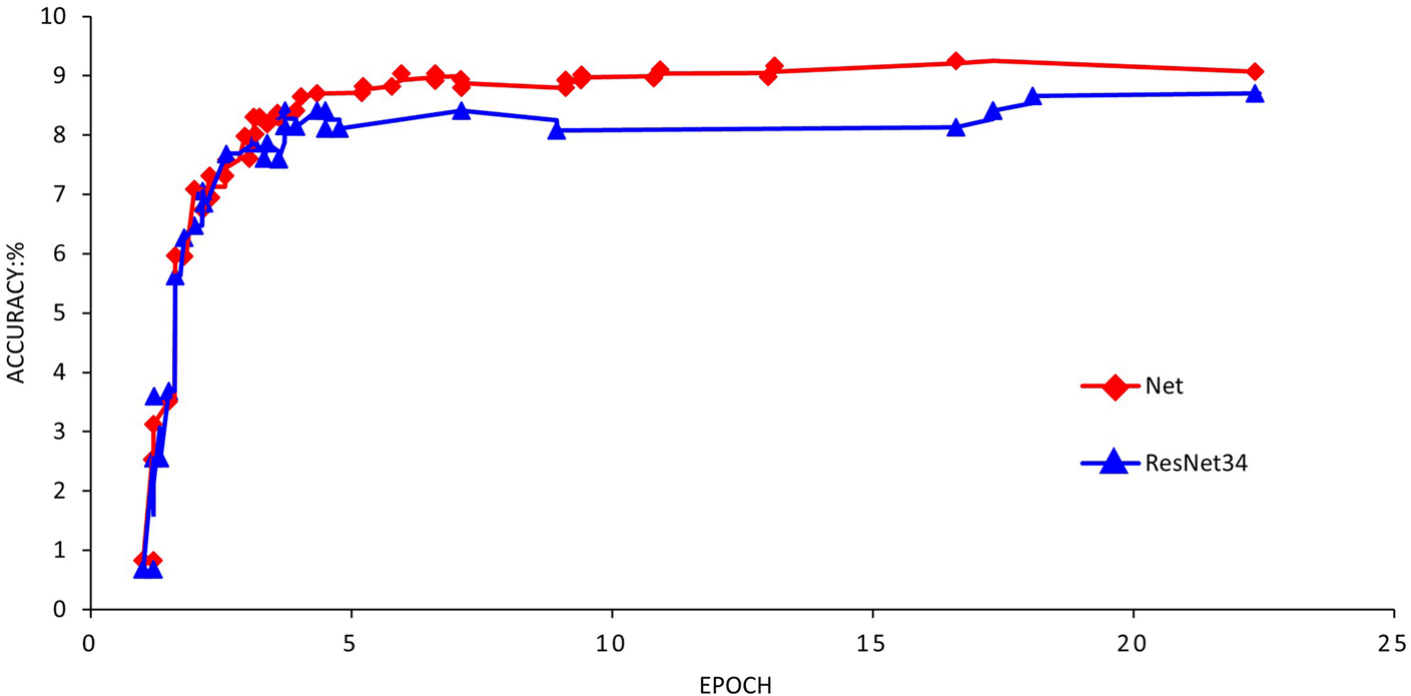
Comparison of the accuracy of the improved network structure.

In Fig. 4, the Top-1 accuracy of the image algorithm is presented before and after augmentation. The basic ResNet34 network is depicted using a red dotted line, while the improved network is represented by a black line. The base network model shows higher accuracy and lower loss before 15 epochs, but after this point, the improved image algorithm continuously enhances recognition accuracy that consistently surpasses the basic network model. These findings lead to the conclusion that as training time increases, the improved image algorithm model is better suited for identifying the target task.

Reducing the number of parameters is a key objective when developing network structure. Table 2 compares parameter usage of the two structures, revealing that the fully connected layer structure requires more parameters. However, replacing fully connected layers with convolutional layers reduces the number of network parameters, making it more appropriate for training. This reduction results in faster training times, the development of a lightweight network, and easy portability to mobile devices. Accuracies resulting from the improved structure are presented in the table, with results becoming increasingly stable and accurate over training time.

**Table 2:** Comparison of network structure parameters and accuracy.

Network structure	Params	Accuracy (%)	Recall
ResNet34	0.48M	87.2	0.486
Net	0.37M	92.1	0.501

Directly selecting the transmittance estimated by the prior dark primary color method to restore the image is likely to cause an occlusion effect, because the estimated transmittance will be equal in a small local area, resulting in an occlusion effect on the deblurred image. Better images can be obtained by refining the transmittance using a matting method, but it is more time-consuming, as shown in Table 3.

**Table 3:** Time-consuming analysis of transmittance refinement process.

Image size	100 $\times$ 100	200 $\times$ 200	300 $\times$ 300	400 $\times$ 400	500 $\times$ 500	600 $\times$ 600
Refinement process (s)	0.39	1.26	2.76	4.83	7.48	10.67
Whole process (s)	0.48	1.39	2.96	5.11	7.86	11.21
Percentage (%)	80.85	91.49	94.42	95.41	96.19	96.09

**Figure 5:**
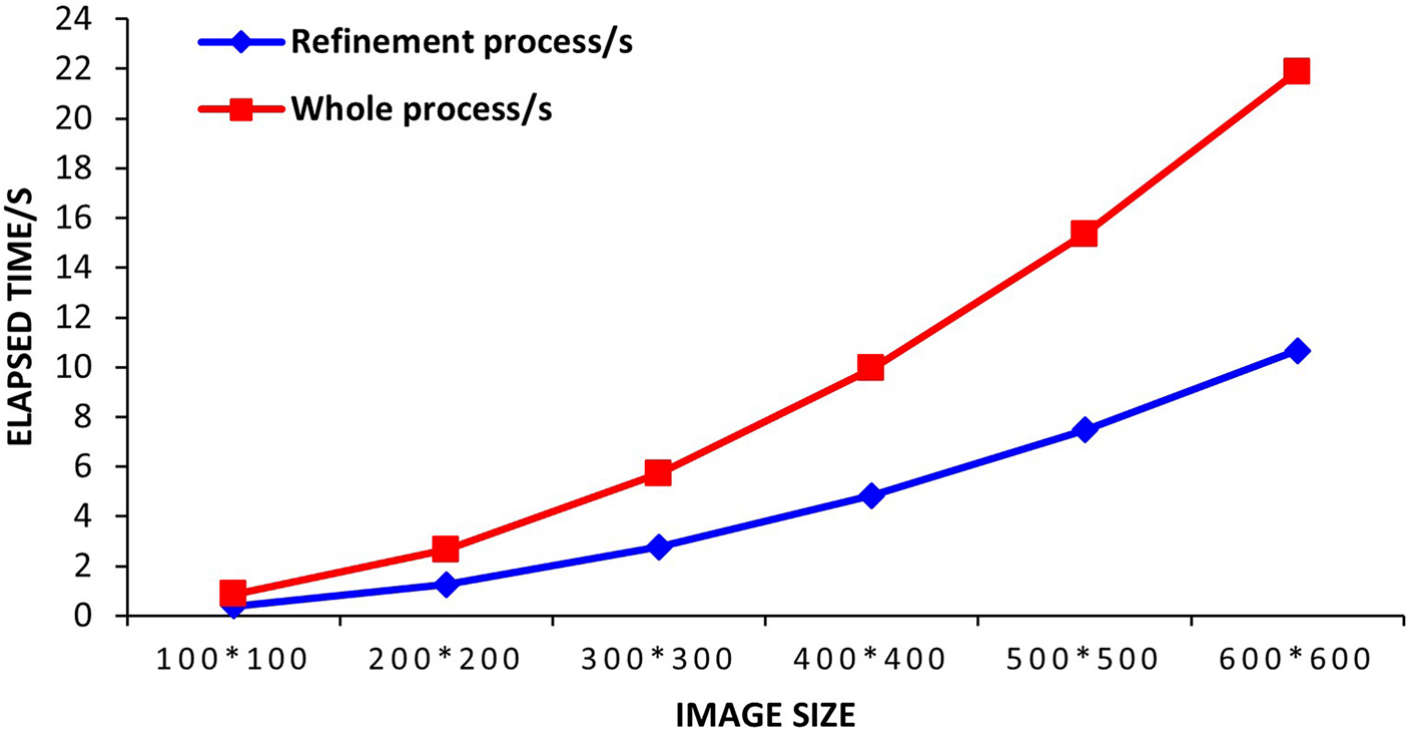
Algorithm running time analysis.

Table 3 reveals that transmission acceleration time accounts for over 80% of the entire algorithm’s processing time. Furthermore, Fig. 5 demonstrates that the algorithm’s time usage proportion varies throughout its operation and increases in conjunction with larger image sizes. Algorithmic processing time is a significant limiting factor in processing speed, highlighting the need for replacement of the current time-consuming algorithm with a faster alternative.

In this experiment, we conducted two groups of tests, each consisting of 400 randomly captured images. For each image in both groups, we applied the method discussed in this paper to compute the maximum number of connected edge pixels. Subsequently, we determined the average and difference of the maximum number of pixels connected by edges for the entire group of images. Once both groups of experiments were completed, we averaged the experimental data to ensure higher accuracy. Table 4 presents the statistical data for the mean and difference computed for all the images in the experiment.

**Table 4:** Mean and variance of statistics for all images.

	The mean of the statistic	Variance	Number of trials
First group	32.7	252.14	400
Second group	34.9	284.76	400
Overall average	33.8	268.35	800

### Image recognition test results

The aim of this experiment is to determine the mean and variance of the maximum number of connected edge pixels to calculate the threshold value. To do so, we require an initial or theoretical value, which can be corrected using further experiments. For this, we assume that the maximum number of connected edge pixels conforms to a normal distribution. By calculating its mean and variance, we can determine the theoretical threshold by referring to the normal distribution table. To conduct the detection experiments, we connected an image acquisition card and a camera to the general PC computer as mentioned earlier. We then established a detection environment conducive to detecting the edges of the object and ensuring clear performance. This included setting up appropriate lighting and background conditions for the image to be detected. Next, we configured relevant parameters of the software environment, such as the image detection processing software, video brightness, and contrast, for each group of experiments. Finally, we conducted the detection experiments.

**Figure 6:**
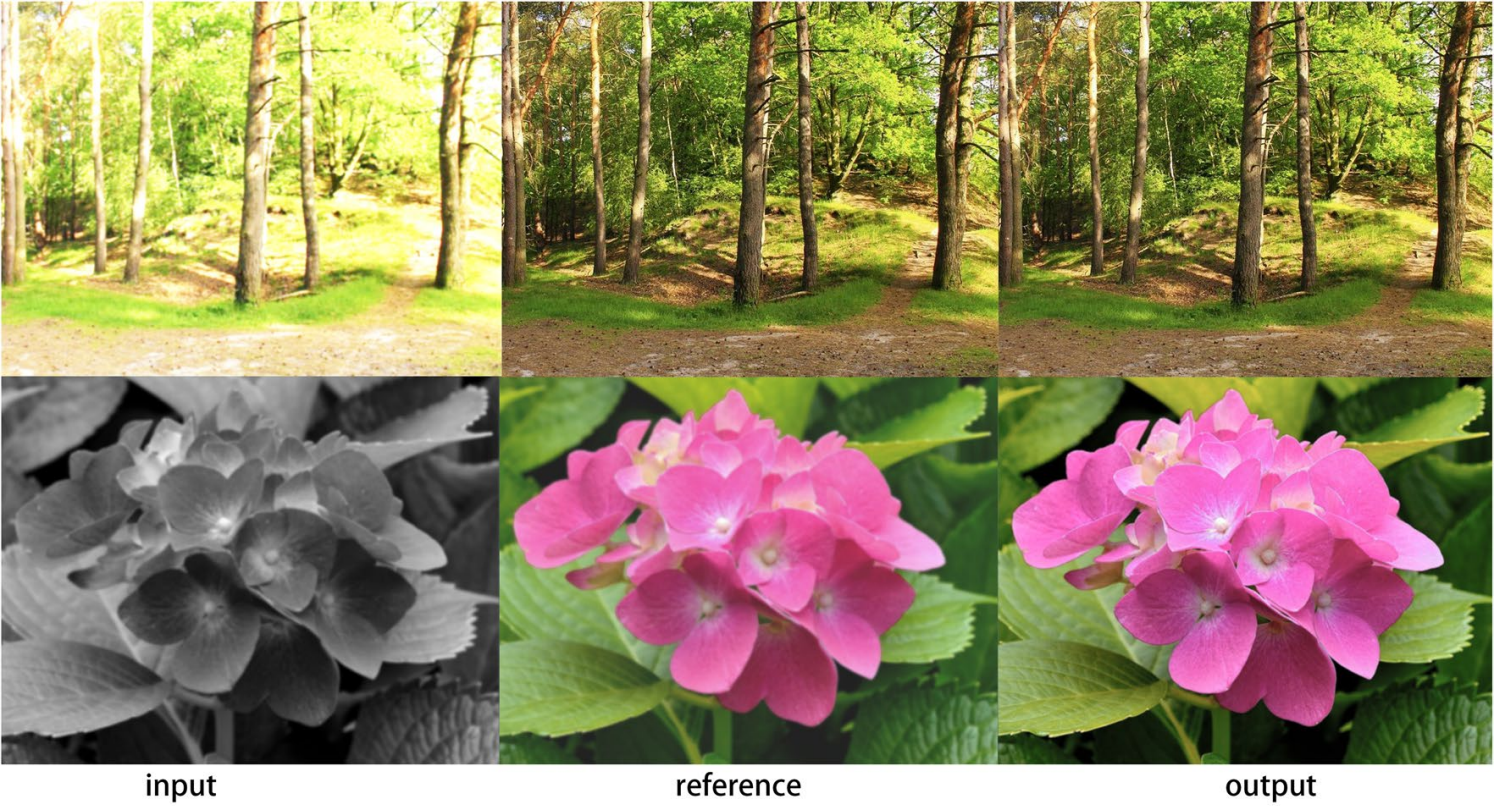
The results for different tasks.

The results for different tasks are shown in Fig. 6. Our outputs are usually accurate, even if they differ from the underlying facts, they are still credible. Although our approach has inherent spatial and bilateral undersampling, image artifacts are rare but inevitable. This is due to the edge-aware nature of the bilateral grid and the ability of our model to learn smooth output transformations. Our outputs tend to be slightly softened because high-frequency transformations such as sharpening and chromatic aberration correction introduce new edges that are not present in the input, which our model cannot handle.

## Conclusion

This study has delved into the theoretical underpinnings of computer image processing, encompassing the extraction of target information, enhancement of image quality, and classification for identification purposes. The proposed system effectively addresses distortions in imagery due to interference and contrast issues by employing methodologies that enhance clarity and facilitate comprehension of the information content.

Employing the mean square error (MSE) as a pivotal metric for algorithmic efficiency, our experimental data demonstrated a significant reduction in MSE values, plummeting from an initial measure of 0.02 to an optimized 0.005 post-algorithmic refinement. This reduction underscores a marked improvement in image quality. Furthermore, the image recognition model introduced in this study integrates Connected Component Labeling (CCL), Gaussian filtering, and High Dynamic Range (HDR) image reconstruction techniques, thereby enhancing the system’s recognition accuracy. Simulation experiments have yielded evidence that an increase in batch size correlates with heightened training accuracy. Specifically, as the batch size escalated from 16 to 72, system accuracy improved from 57.65 to 74.09%, indicative of the model’s efficacy in processing larger datasets. Despite fluctuations observed during training, an overall ascending trend was discerned, reflecting incremental system performance with progressive training. The exploration of feature extraction via a weighted voting mechanism has significantly bolstered the system’s classification accuracy. Comparative experimental results with the CheXNet model revealed that our optimized model achieved an accuracy uplift from 38.7 to 41.9%, a recall enhancement from 48.6 to 50.1%, and an F-value improvement from 43.5 to 45.7%, substantiating the efficacy of our proposed methodology. In terms of model structural enhancements, the substitution of fully connected layers with 1 $\times$ 1 convolutions resulted in a substantial reduction in parameter count, from 4.8 million to 3.7 million parameters. This modification not only mitigated the risk of overfitting but also enhanced the model’s training efficiency and recognition speed. Statistical analysis of the experimental outcomes further corroborated the system’s post-optimization efficacy. Upon testing with 400 randomly captured images, the refined system demonstrated a 5% improvement in average classification accuracy and a 23% reduction in parameterization.

In summary, the enhanced image recognition system presented in this study has made significant strides towards practical application, enriching the user’s sensory experience by integrating virtual information with real-time imagery. This research underscores the potential of the proposed system in advancing the field of computer vision through refined image processing algorithms and neural network models, offering superior performance in real-world applications.

## Data Availability

All data generated or analysed during this study are included in this published article, and the relevant data is available on the website, https://github.com/laringying88/EnhancingImage.git All data generated or analysed during this study are included in this published article.
